# Global climate change‐driven impacts on the Asian distribution of *Limassolla* leafhoppers, with implications for biological and environmental conservation

**DOI:** 10.1002/ece3.70003

**Published:** 2024-07-18

**Authors:** Weiwei Ran, Jiajia Chen, Yuanqi Zhao, Ni Zhang, Guimei Luo, Zhibing Zhao, Yuehua Song

**Affiliations:** ^1^ School of Karst Science Guizhou Normal University Guiyang China; ^2^ State Engineering Technology Institute for Karst Desertification Control Guiyang China; ^3^ School of Food Science and Engineering Guiyang University Guiyang China

**Keywords:** biodiversity, global climate change, habitat suitability, long‐term changes, macroecology, species distribution models

## Abstract

Knowing the impacts of global climate change on the habitat suitability distribution of *Limassolla* leafhoppers contributes to understanding the feedback of organisms on climate change from a macroecological perspective, and provides important scientific basis for protecting the ecological environment and biodiversity. However, there is limited knowledge on this aspect. Thus, our study aimed to address this gap by analyzing Asian habitat suitability and centroid shifts of *Limassolla* based on 19 bioclimatic variables and occurrence records. Selecting five ecological niche models with the outstanding predictive performance (Maxlike, generalized linear model, generalized additive model, random forest, and maximum entropy) along with their ensemble model from 12 models, the current habitat suitability of *Limassolla* and its future habitat suitability under two Shared Socio‐economic Pathways (SSP1‐2.6 and SSP5‐8.5) in the 2050s and 2090s were predicted. The results showed that the prediction results of the five models are generally consistent. Based on ensemble model, 11 potential biodiversity hotspots with high suitability were identified. With climate change, the suitable range of *Limassolla* will experience both expansion and contraction. In SSP5‐8.52050s, the expansion area is 118.56 × 10^4^ km^2^, while the contraction area is 25.40 × 10^4^ km^2^; in SSP1‐2.62090s, the expansion area is 91.71 × 10^4^ km^2^, and the contraction area is 26.54 × 10^4^ km^2^. Furthermore, the distribution core of *Limassolla* will shift toward higher latitudes in the northeast direction, and the precipitation of warmest quarter was found to have the greatest impact on the distribution of *Limassolla*. Our research results supported our four hypotheses. Finally, this research suggests establishing ecological reserves in identified contraction to prevent habitat loss, enhancing the protection of biodiversity hotspots, and pursuing a sustainable development path with reduced emissions.

## Graphical abstract

This study conducted a comprehensive analysis of the distribution of *Limassolla* based on different Shared Socio‐economic Pathways and climate scenarios, incorporating theories and methods from insect ecology, environmental ecology, and biogeography. The results indicated that *Limassolla* is mainly distributed in East Asia, South Asia, and Southeast Asia, and the precipitation of warmest quarter has been found to have the greatest impact on the distribution of *Limassolla*. With climate change, the suitable range of *Limassolla* will experience both expansion and contraction, with the distribution core shifting toward higher latitudes in the northeast direction.

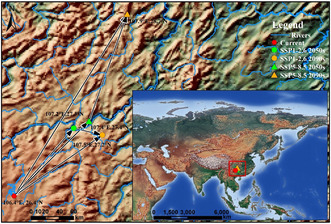

## 1 INTRODUCTION

The genus *Limassolla* Dlabola, 1965 leafhoppers (Hemiptera: Cicadellidae: Typhlocybinae) are a group of phytophagous insects. These leafhoppers typically inhabit host plants, which include persimmon trees, southern sour jujube trees, bayberry trees, Chinese sumac, mulberry, and other broad‐leaved plants. They generally feed on the leaves, have limited dispersal capabilities, and are adept at jumping. Forty‐nine species of this genus have been reported worldwide, mainly distributed in the Oriental, Palaearctic, and Afrotropical regions (Oh et al., 2020; Yuan et al., 2020; Zhou et al., 2020). They are greatly influenced by climatic conditions, with mostly species overwintering as eggs or adults and continuing their growth and development in favorable climatic conditions without a true dormancy period (Zhang, 1990). Due to their sensitivity to environmental changes, they play a significant role as indicator organisms in ecological environment protection, the relationship between organisms and environmental changes, and ecosystem stability (McGeoch, 1998; Zhen‐Qiang et al., 2015). Therefore, we can associate the distribution changes in leafhoppers with biodiversity and environmental protection. However, because of their sensitivity to environmental changes, in addition to natural predators such as spiders, ladybugs, and lacewings, they are also vulnerable to potential threats from climate change.

Nowadays, climate change is widely recognized as the most significant threat facing humanity today (Alkhalifah et al., 2023; Tiedje et al., 2022). Observations indicate that there have been changes in extreme weather and climate events since the mid‐20th century. These changes include a decline in cold temperature extremes, a rise in warm temperature extremes, accelerated sea‐level rise, and an amplified occurrence of heavy precipitation events in various areas (Scott et al., 2021; Skendžić et al., 2021). However, climate plays a vital role in shaping biotic systems, it affects various aspects such as the fitness of individuals, population dynamics, species distribution and abundance, as well as the structure and functioning of ecosystems (Addo‐Bediako et al., 2000; Parmesan et al., 2000). The response of species to climate change is commonly analyzed within a framework: Species and populations have the capability to shift their distribution and follow favorable environments; however, if suitable habitat is unavailable or if the species lacks the ability to migrate, adaptation becomes crucial for their survival, as extinction becomes a potential outcome (Kellermann & Van Heerwaarden, 2019). The results so far indicate that climate change has had a significant impact on insects (Halsch et al., 2021). Insects represent the majority of animal diversity on earth, understanding their response to climate change remains a significant challenge in the field of climate change biology (Kellermann & Van Heerwaarden, 2019). Therefore, understanding and predicting how insects respond to climate change in different climate scenarios is still important.

Scenarios play a critical role in climate change research and assessment by facilitating the understanding of the long‐term impacts of near‐term decisions and allowing researchers to examine various potential futures within the framework of inherent uncertainties. Shared Socio‐economic Pathways (SSPs) aims to facilitate climate change research and policy analysis, spanning a wide array of scenarios that cover diverse challenges related to mitigation and adaptation to climate change (Riahi et al., 2017). Ecological niche models (ENMs), also referred to as species distribution models (SDMs), habitat suitability models, and bioclimatic envelope models, offer a method to evaluate the impacts generated by climatic factors by connecting species occurrence data with environmental variables and projecting potential effects under realistic future scenarios (Briscoe et al., 2019; Franklin, 2023). ENMs relies on the ecological niche concept, where the ecological niche is defined as the environmental conditions that support the growth and survival of one species (Mugo & Saitoh, 2020). And ENMs have the ability to map habitats, generate dependable and replicable data, and provide valuable information for making informed decisions (Martínez et al., 2015; Sofaer et al., 2019). However, for ENMs, there are numerous modeling methods, and the ongoing challenge lies in evaluating the relative predictive performance of these different methods (Tsoar et al., 2007). Utilizing multi‐algorithm approaches allows for the comparison of outputs generated by various algorithms, leading to the derivation of more robust results (Mugo & Saitoh, 2020). The ensemble model (EM) can combine the results of multiple model methods to obtain more accurate prediction results than a single model (Naimi & Araújo, 2016). The sdm (Naimi & Araújo, 2016), Biomod (Thuiller et al., 2009), mopa (Iturbide, 2015), ModEco (Guo & Liu, 2010), and OpenModellor (De Souza Muñoz et al., 2011) are several commonly used software packages for EM, each with its advantages and limitations, depending on factors such as user experience and preferences (Mugo & Saitoh, 2020).

Currently, the use of ENMs in leafhopper research includes studies such as Jiang et al. (2022) utilized the Maxent model to predict the potential suitable areas for tea green leafhoppers *Matsumurasca onukii* and *Empoasca vitis* in China, Wei et al. (2023) predicted the distribution of *Cicadella viridis* in China based on the MaxEnt. However, the above studies did not link the distribution changes of leafhoppers to biodiversity and environmental protection. Until now, no studies have been conducted on the leafhopper genus *Limassolla* Dlabola, 1965 based on ENMs. Therefore, it is urgent to study the response of the *Limassolla* to climate change using ENMs, which is crucial for biodiversity and environmental conservation. To fill this knowledge gap, we combined theories and methods from insect ecology, environmental ecology, and biogeography in this study. We selected five ENMs with the best prediction performance and established an ensemble model (EM) to predict the Asian habitat suitability of *Limassolla* under different climate scenarios. This study aimed to answer the following scientific questions: (1) What is the most important bioclimatic variable influencing the distribution of the *Limassolla*? (2) What is the predicted future conservation status due to climate change? (3) Will the future Asian habitat of the *Limassolla* contract or expand under climate change? (4) Will the *Limassolla* also move northward/to higher latitudes like other insects or animals under climate change? (5) What are the implications for biological and environmental conservation? Additionally, the study makes the following hypotheses: 1. Temperature and precipitation both influence *Limassolla*'s distribution. 2. Due to climate change, the future main distribution areas should generally align with the current distribution, but there may be habitat loss. 3. Under climate change, *Limassolla*'s distribution will experience both expansion and contraction. 4. To adapt to global warming, *Limassolla* will move to higher latitude areas with suitable temperatures.

## 2 MATERIALS AND METHODS

### 2.1 Occurrence records

As of March 10, 2023, we have collected a total of 159 extant records from 49 species of *Limassolla* from an monograph (Song & Li, 2014), 3I Interactive Keys and Taxonomic Databases (http://dmitriev.speciesfile.org/), and published literature. Typically, the spatial distribution of organisms are shaped by the ecological influences and the biogeographical history associated with a taxon, so all collected occurrence records are included in this study to project the potential distribution range of the genus *Limassolla* in Asia (Liu et al., 2022). After removing incorrect locations and randomly eliminating duplicate locations, there are 123 valid distribution points remaining. The precise latitude and longitude were obtained using the Google platform with the wgs84 coordinate system, and then confirmed and corrected by Google Earth (Mudereri et al., 2021). When spatial clusters of localities are present, models often suffer from overfitting toward environmental biases, leading to decreased ability to predict spatially independent data, and inflated model performance values (Boria et al., 2014). Therefore, in order to avoid these issues, the “Spatially Rarefy Occurence Data for SDMs” tool in SDM toolbox 2.0 was used to sparse the spatial distribution points of species, and the resolution to rarefy data was set to 10 km (Aidoo et al., 2023). Finally, 114 qualified modeling distribution point data from 44 species were obtained (Table S1).

### 2.2 Bioclimatic variables

In this study, 19 bioclimatic variables (Table S2) with a spatial resolution of 5 min, suitable for national to global scales, were used from WorldClim version 2.1 (https://worldclim.org/data/index.html) to predict the Asian potential distribution and habitat suitability of *Limassolla* (Chen et al., 2021; Gu et al., 2023; Zhang et al. 2022b; Zhou et al., 2023). This version was released in January 2020 and has been successfully applied to species distribution predictions (Aidoo et al., 2023; Elith & Leathwick, 2009; Jin et al., 2022; Zhang et al. 2022a). In addition, some researchers have shown that the WorldClim dataset offers an adequate basis of data for SDM analysis when exclusively employing bioclimatic variables, without the inclusion of other climatological variables (Merkenschlager et al., 2023).

The current 19 bioclimatic variables are derived from the average values of data from 1970 to 2000 (Jendritzki et al., 2023; Ramasamy et al., 2022). For future bioclimatic variables, they are based on Coupled Model Intercomparison Project Phase 6 (CMIP6) downscaled future climate projections (Eyring et al., 2016), and are from two periods (2050s and 2090s) under SSP1‐2.6 and SSP5‐8.5 scenarios. The provided data for the two future prediction periods are averages of monthly values that were recorded over 20‐year time frames (2041–2060 and 2081–2100) (Fick & Hijmans, 2017), reflecting both nearer future and longer term climatic conditions (Jendritzki et al., 2023; Riahi et al., 2017; Zhang et al., 2019). Moreover, the SSP1‐2.6 scenario presents an optimistic outlook for global sustainable development in the future, whereas the SSP5‐8.5 scenario represents the most adverse emission scenario, which ignores the mitigation of climate change and mandates extensive adaptation measures (Riahi et al., 2017). By considering these two scenarios, which will influence species distribution to varying degrees, we can evaluate the distribution changes of *Limassolla* to different emission pathways and explore a range of possible outcomes (Jamal et al., 2021; Rogelj et al., 2018; Xu et al., 2022). The global climate model we chose is the Model for Interdisciplinary Research on Climate (MIROC‐6), which is a newly developed climate model that upgrades in the physical parameterizations of all sub‐modules (Tatebe et al., 2019).

Due to the potential presence of multicollinearity among bioclimatic variables, which can lead to unstable and overfitting models (Hebbar et al., 2022), variable selection is crucial for determining modeling accuracy (Aidoo et al., 2022). In this study, the following steps were taken for the 19 bioclimatic variables: (1) The variables were simultaneously inputted with 114 species distribution records into Maximum Entropy Species Distribution Modeling, Version 3.4.4 (https://biodiversityinformatics.amnh.org/open_source/maxent/) to establish initial model and calculate the contribution of each variable to the model (Aidoo et al., 2022; Khan et al., 2022; Phillips et al., 2017); (2) ArcGIS's spatial analysis functionality was utilized to extract the corresponding bioclimatic variables for the distribution points, and IBM SPSS Statistics Version 22 was employed to calculate the Pearson correlation coefficients between the climate variables (Mao et al., 2022); (3) If the correlation coefficient between two bioclimatic variables exceeded 0.7 (Aidoo et al., 2023; Azrag et al., 2022; Ramos et al., 2019) (Figure S1), the variable with a higher percentage contribution in the initial model was retained (Mao et al., 2022; Yang et al., 2013). Through these steps, eight qualified bioclimatic variables were selected for building the final models (Table S2).

### 2.3 Modeling process

In order to mitigate the impact of varying modeling techniques on the uncertainty in the modeling results (Guo et al., 2019), we utilized “sdm” package in the R software (Naimi & Araújo, 2016) to select the top five models based on the average area under the receiver operating characteristic curves (AUC) from 12 commonly used ecological niche models for predicting the habitat suitability of *Limassolla*. The 12 commonly used ecological niche models include random forest (RF) (Breiman, 2001), generalized additive model (GAM) (Hastie & Tibshirani, 2014), Maxlike (Royle et al. 2012), generalized linear model (GLM) (Friedman et al., 2010), maximum entropy (Maxent) (Phillips et al. 2006), flexible discriminant analysis (FDA) (Hastie et al., 1994), multivariate adaptive regression splines (MARS) (Moisen & Frescino, 2002), GLMNET (Yuan et al., 2011), support vector machines (SVM) (Kindt, 2018), Domain (Carpenter et al., 1993), classification and regression trees (CART) (Hu et al., 2011), and Bioclim (Booth et al., 2014) (Figure S2). The “sdm” package integrates various ecological niche and machine learning models into a single platform, facilitating parallel execution (Naimi & Araújo, 2016). It adopts object‐oriented, reproducible, and extensible approaches within the R environment to enhance efficiency and reduce redundancy in the research paper (Naimi & Araújo, 2016). The model parameters were set as follows:

1. The “sdmData” function was used to generate 1000 pseudo‐absence records (Azrag et al., 2022; Mudereri et al., 2021; Puchałka et al., 2021), which were combined with 114 presence‐only records of *Limassolla* for modeling. This approach ensured a balanced dataset for training the model and reduced the potential bias introduced by using only presence records. And the 114 distribution records were adequate, as the accuracy approached its maximum level even with 50 data points (Ran et al., 2023; Stockwell & Peterson, 2002).
2. We partitioned the distribution data into a test set comprising 25% of the data and a training set containing the remaining 75% with a 10‐fold cross‐validation approach (Adhikari et al., 2023; Liu et al., 2022; Roberts et al., 2017). We set the maximum number of iterations to 5000 (Wang et al., 2020). This provided an adequate opportunity for the model to optimize its performance and reach a stable state during the training process.
3. Given the potential variations in results among different model algorithms, we employed an EM using the true skill statistical (TSS) weighted average approach (Guo et al., 2019; Mugo & Saitoh, 2020). This method combined the results of the five selected models (Maxlike, GLM, GAM, RF, and Maxent). The EM demonstrated superior predictive performance compared with the individual model outputs, enhancing the overall accuracy and reliability of the predictions (Hao et al., 2019; Naimi & Araújo, 2016).

### 2.4 Model evaluation and accuracy

Five evaluation parameters, namely AUC, correlation (COR), TSS, explained deviance between the calibrated and evaluated values, and Kappa, were utilized to measure the goodness of fit of the model (Allouche et al., 2006). In general, the AUC values range from 0 to 1, with a value of 1 indicating the highest level of model performance. This metric is commonly used to assess the accuracy and predictive power of models (Fourcade et al., 2018; Freer et al., 2022; Proosdij et al., 2016; Zhang et al., 2018), and a model with an AUC value of ≥0.7 is considered to have a high level of predictive performance (Kindt, 2018; Mudereri et al., 2021). The COR represents the correlation between the observations in the presence–absence dataset and the corresponding predictions. It provides insights into the level of agreement between the observed and predicted values (Elith et al., 2006). The TSS is a measure that evaluates both the specificity and sensitivity of a model while considering omission and commission errors (Allouche et al., 2006). It ranges from −1 to +1, where a value of +1 indicates perfect discrimination by the model. Similar to the TSS, the kappa statistic also falls within the range of −1 (indicating poor agreement) to +1 (representing perfect prediction) (Allouche et al., 2006).

### 2.5 Potential spatiotemporal distribution and centroid changes

Using the ArcGIS software, the habitat suitability was categorized into five distinct categories: High suitability (0.8–1), optimum suitability (0.6–0.8), moderate suitability (0.4–0.6), low suitability (0.2–0.4), and unsuitable habitat (0.0–0.2) (Ab Lah et al., 2021; Maruthadurai et al., 2023; Ramasamy et al., 2022). This division allowed for a more structured representation of the habitat suitability levels and facilitated a clearer understanding of the distribution patterns and ecological preferences of the population.

“Distribution Changes Between Binary SDMs” and “Centroid Changes (Lines)” in the SDM toolbox v2.5 (Brown, 2014) were used to analyze the potential spatiotemporal distribution changes in *Limassolla*. The distribution range change maps were generated by comparing the ensemble habitat suitability map for the present climate conditions with the maps projected for 2050 and 2090 under SSP1‐2.6 and SSP5‐8.5 climate scenarios. The comparison was conducted after converting the current and future potential distributions of the *Limassolla* into two categories: unsuitable (with suitability values less than 0.20) and suitable (with suitability values greater than 0.20) (Liu et al., 2022; Ramasamy et al., 2022). The output results of “Distribution Changes Between Binary SDMs” fall into four categories: range expansion, no occupancy (representing absence in both), no change (representing presence in both), and range contraction.

Furthermore, the centroid (species distribution core) analysis can be used to describe the spatial distribution of geographical objects, and the movement or displacement of these objects over a specific time period can be characterized by changes in their respective centroids (Jin et al., 2022; Phillips et al. 2006). The focus of this analysis is to summarize the core shifts in distributional ranges of multiple species. This analysis involves reducing the distribution of each species to a single central point, known as a centroid, and generating a vector file that represents the magnitude and direction of predicted change over time. Therefore, by using “Centroid Changes (Lines)” in the SDM toolbox v2.5 (the ‘Input Raster Format’ is ‘TIFF’), we can analyze the changing trend of *Limassolla*'s Asian distribution core under climate change.

Finally, by assessing the contraction and expansion of the suitable habitat of *Limassolla* leafhoppers, as well as the direction and magnitude of movement of the centroid, we can infer the implications for biological and environmental conservation.

## 3 RESULTS

### 3.1 Accuracy and selection of models

**FIGURE 1:**
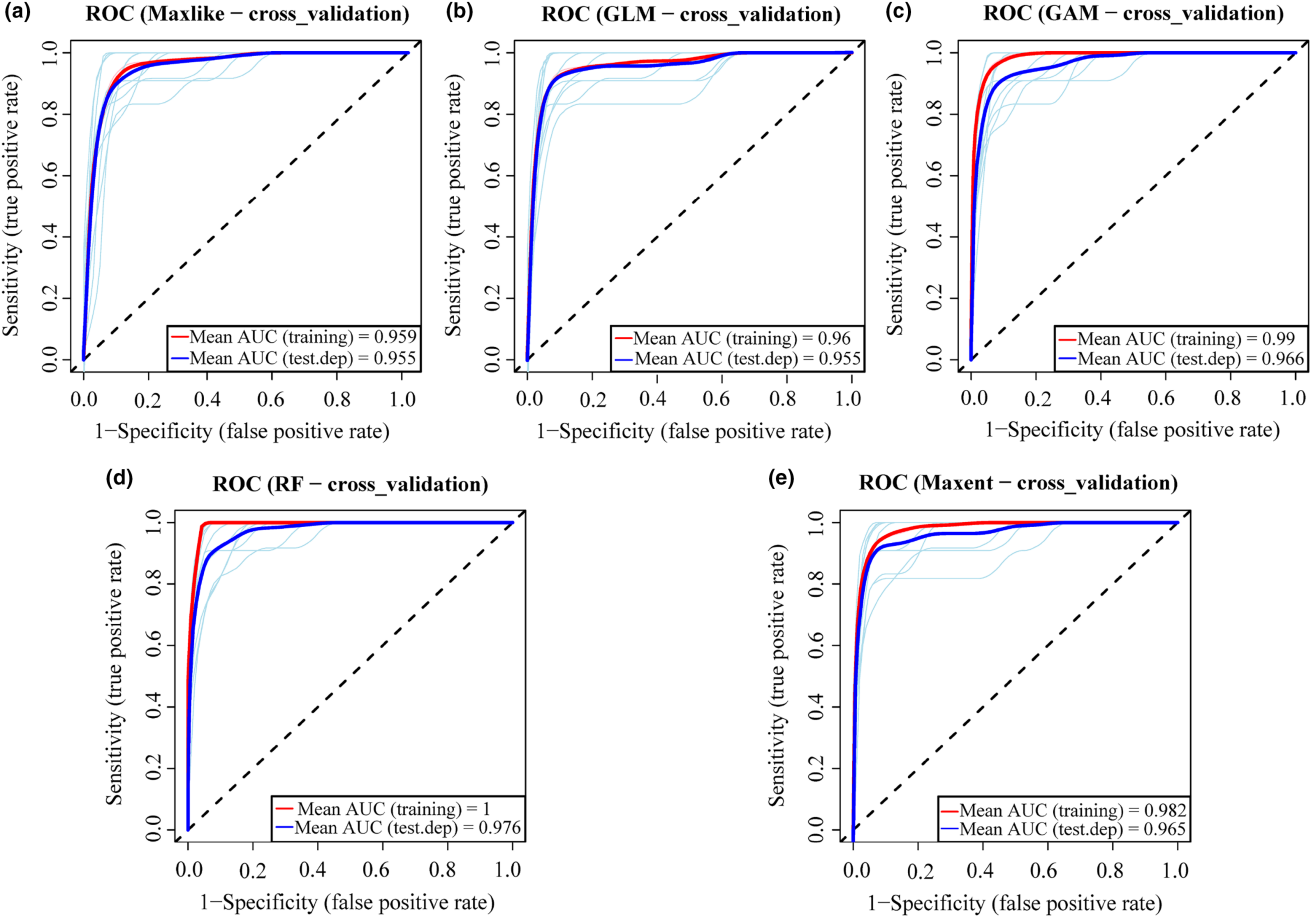
The receiver operating curves (ROCs) of the five algorithms that are selected to predict habitat suitability of the *Limassolla*, with (a) Maxlike, (b) GLM, (c) GAM, (d) RF, and (e) Maxent.

In this study, the 10‐fold cross‐validation approach were used, which is a good choice for evaluating the accuracy of the model (Wong & Yeh, 2020). This approach involves dividing data into multiple distinct subsets, which are then utilized alternately for calibrating and validating models. The evaluation parameter values for all 12 models are shown in Figure S2. Since the AUC value is the most used evaluation parameter, the top five models based on the AUC test values were selected: Maxlike, GLM, GAM, RF, and Maxent (Figure S2b, Figure 1). These five models have excellent predictive performance as indicated by the following ranges: 1 ≥ AUC training ≥ 0.96, 0.98 ≥ AUC test ≥ 0.96, 0.80 ≥ COR ≥ 0.73, 0.90 ≥ TSS ≥ 0.86, 0.37 ≥ Deviance ≥ 0.24, and 0.76 ≥ Kappa ≥ 0.68 (Table S3). This implies that all five models demonstrate excellent predictive capabilities.

### 3.2 Contribution and importance of bioclimatic variables

**FIGURE 2:**
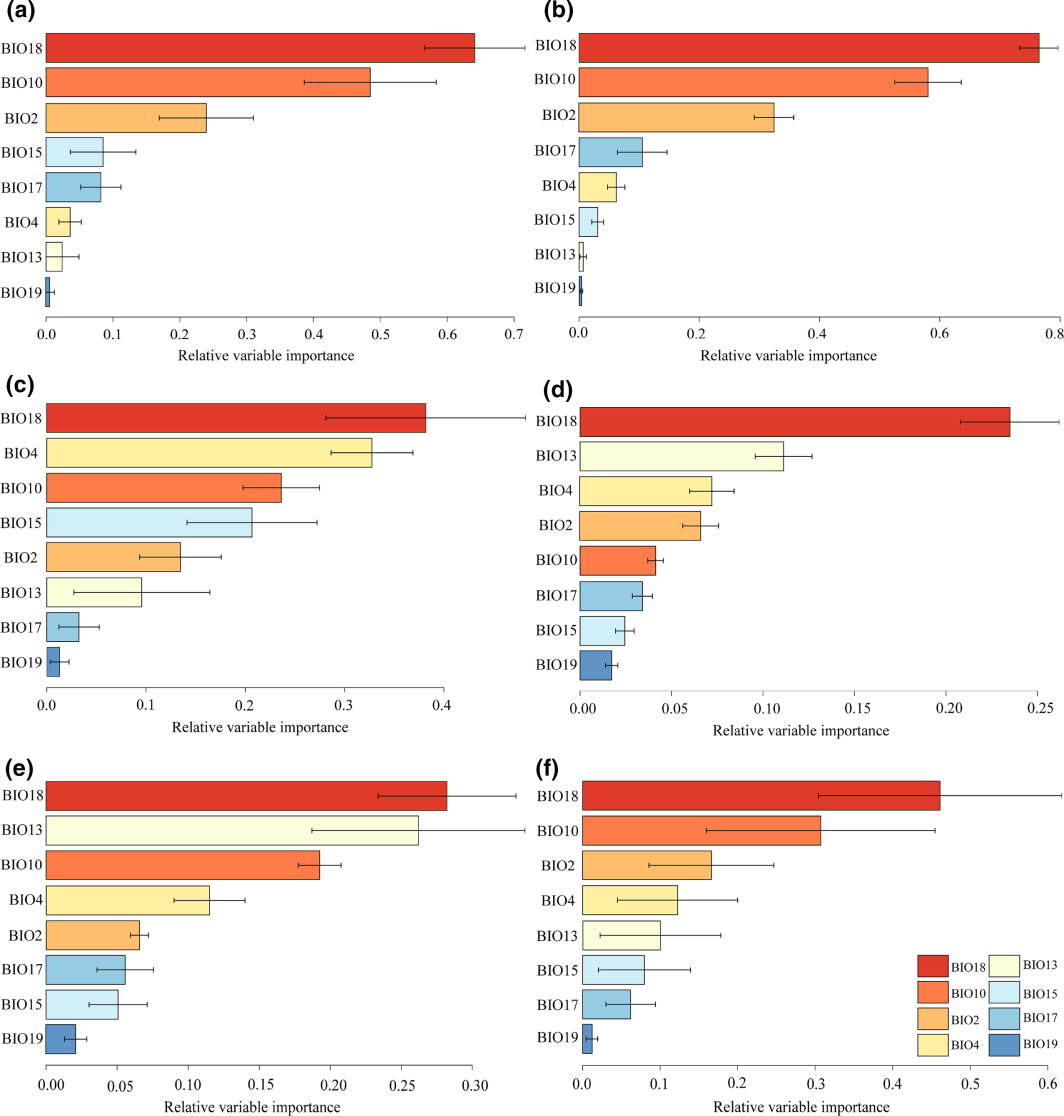
Relative variable importance for predicting the habitat suitability of the *Limassolla* using (a) Maxlike, (b) GLM, (c) GAM, (d) RF, (e) Maxent, and (f) Combined (averaged).

According to the three steps of variable selection mentioned in the Materials and Methods: 2.2 Section, the detailed variable selection method is as follows: The correlation between BIO3 and BIO1 is greater than 0.7 (Figure S1), but the contribution rate of BIO3 is higher than that of BIO1 (Table S2), so BIO3 is retained. However, the correlation between BIO4 and BIO3 is also greater than 0.7 (Figure S1), and the contribution rate of BIO4 is higher than that of BIO3 (Table S2); thus, the previously retained BIO3 is removed and BIO4 is kept instead. This process is continued, ultimately resulting in the selection of eight variables for modeling. The selected eight bioclimatic variables from the 19 options are mean diurnal range (BIO2), temperature seasonality (BIO4), mean temperature of warmest quarter (BIO10), precipitation of wettest month (BIO13), precipitation seasonality (BIO15), precipitation of driest quarter (BIO17), precipitation of warmest quarter (BIO18), and precipitation of coldest quarter (BIO19) (Table S2). Among these variables, BIO18 contributes the most to the model, accounting for 40.38% of the contribution. Moreover, the Maxlike, GLM, GAM, RF, and Maxent models all show that BIO18 is the most important variable (Figure 2), indicating its significant impact on the spatiotemporal distribution pattern of the *Limassolla*. The average variable importance from these five models (Figure 2f) indicates that BIO18, BIO10, BIO2, and BIO4 are the top four variables influencing the spatiotemporal distribution pattern of the *Limassolla*. Therefore, temperature and precipitation are both crucial for the occurrence of the *Limassolla*, which validates our proposed hypothesis 1.

### 3.3 Current Asian distribution of *Limassolla*

**FIGURE 3:**
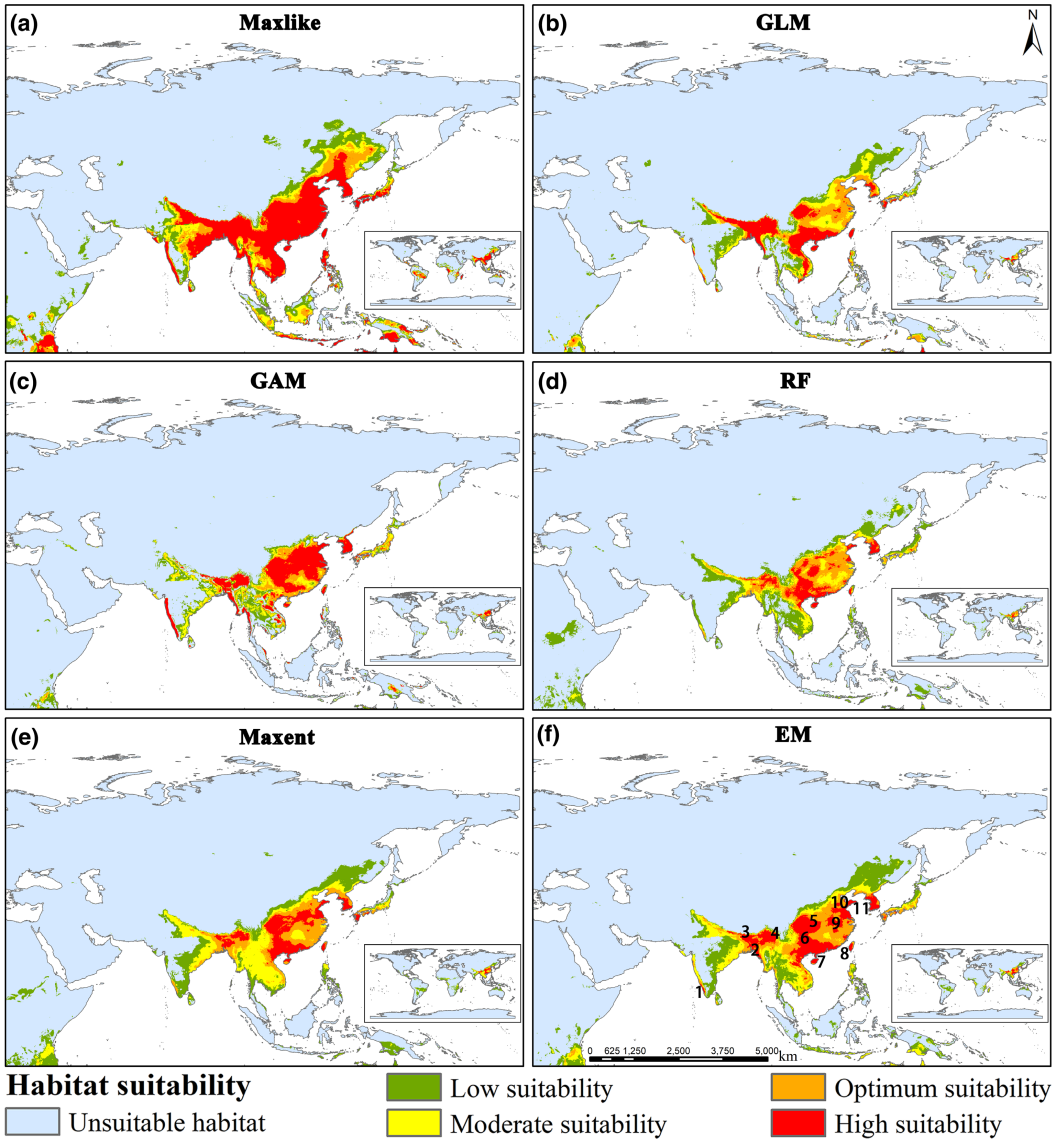
Habitat suitability of *Limassolla* under current climate scenario using (a) Maxlike, (b) GLM, (c) GAM, (d) RF, (e) Maxent, and (f) EM. Numbers represent the biodiversity hotspots with high habitat suitability.

The predictions of the five models show slight differences, but all indicate that the distribution of the *Limassolla* is primarily in East Asia, South Asia, and Southeast Asia (Figure 3). Specifically, the predictions from all five models show that the *Limassolla* is mainly distributed in the southeast of the 400 mm equivalent precipitation line in China, as well as in North Korea, South Korea, and Japan; India, Bangladesh, Bhutan, and Nepal; Myanmar, Laos, Vietnam, Thailand, Cambodia, and the Philippines. (Figure 3). However, the Maxlike model shows more high‐suitability habitats than the other four models (Figure 3a). GAM, RF, and Maxent models show that Thailand and Cambodia have very few high suitability areas (Figure 3c–e). Based on the EM prediction, high suitability areas are mainly distributed in China's Hainan, Guangdong, Guangxi, Guizhou, Sichuan, Chongqing, Hubei, Anhui, Jiangsu, Shandong, and Taiwan; southern parts of North Korea; South Korea; northern regions of Myanmar and Vietnam; central areas of Laos; northeastern parts of India and Bangladesh (Figure 3f). The areas of high, optimum, moderate, and low suitability zones in Asia are 153.39 × 10^4^ km^2^, 159.42 × 10^4^ km^2^, 163.43 × 10^4^ km^2^, and 245.76 × 10^4^ km^2^, respectively (Table S4). Furthermore, we identified 11 high‐suitability areas which can serve as potential biodiversity hotspots (Liu et al., 2022) (Figure 3f). These 11 areas, respectively, are the southeast of the Western Ghats, Arakan Yoma, the southern Himalayas, the Hengduan Mountains, Daba Shan, the Yunnan‐Guizhou Plateau, Wu‐chih Mountains, Central Range, Ta‐pieh Mountains, Mount Tai, and the Sobaek Mountains. The predicted results are nearly consistent with the actual occurrence points, demonstrating the accuracy of the model predictions.

### 3.4 Future potential Asian distribution of *Limassolla*

**FIGURE 4:**
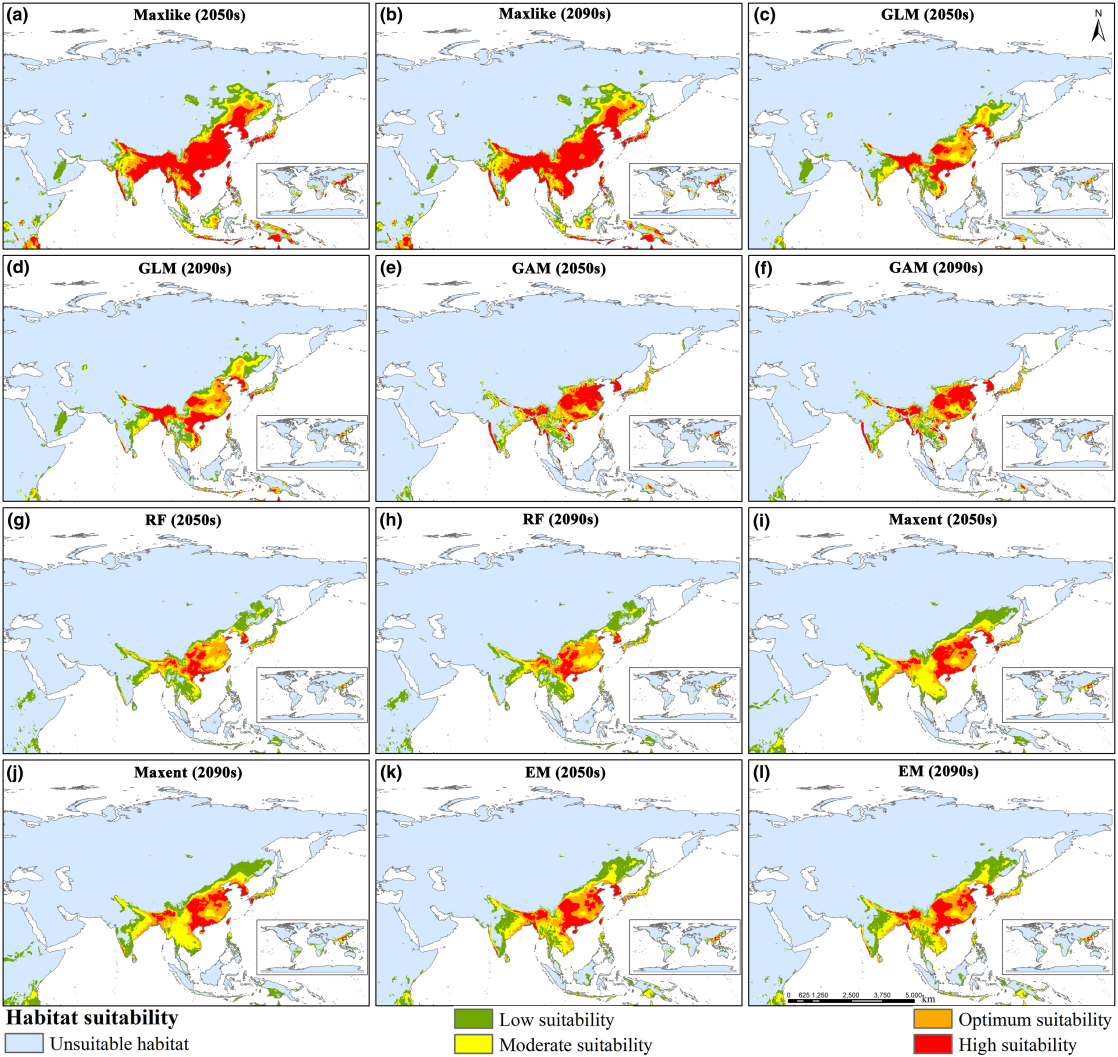
Habitat suitability of *Limassolla* under future SSP1‐2.6 climate scenario using Maxlike, GLM, GAM, RF, Maxent, and EM.

In the SSP1‐2.6 climate scenario, the differences in predicted results among the five models and their EM are not significant in the 2050s and 2090s. Their main distribution ranges are largely consistent with current climate conditions (Figures 3 and 4). However, based on Maxlike predictions, the suitable habitats in Russia have significantly increased, while the low suitability areas in China have increased, and the high suitability areas in Thailand have decreased (Figure 4a,b). The GLM model predicts an increase in suitable areas in Russia, Myanmar, and Thailand, an increase in optimum and moderate suitable areas in northeastern China, and a decrease in suitable areas in northwestern India (Figure 4c,d). Suitable areas in northwestern India decrease for GAM, while moderate suitability areas decrease in Thailand (Figure 4e,f). RF shows an increase in low suitability areas in northeastern China and a decrease in suitable areas in northwestern India (Figure 4g,h). The Maxent predicts a decrease in suitable areas in northwestern India, an increase in high, optimal, and moderate suitability areas in northeastern China, and an increase in suitable areas in Russia (Figure 4i,j). EM, through weighted averaging of the predictions from the five models mentioned, shows a significant increase in suitability areas near the Russian region close to Heilongjiang Province in China. The moderate suitability areas in the northeastern region of China also noticeably increase, while decreasing in the northwest region of India (Figure 4k,l). Under the SSP5‐8.5 climate scenario, compared with SSP1‐2.6, the distribution ranges of the suitable areas are generally consistent, with an overall increase in total area, but there are differences in local changes (Table S4, Figure S3 and Figure 4).

### 3.5 Potential Asian spatiotemporal distribution and centroid changes of *Limassolla* based on ensemble model under climate change

**FIGURE 5:**
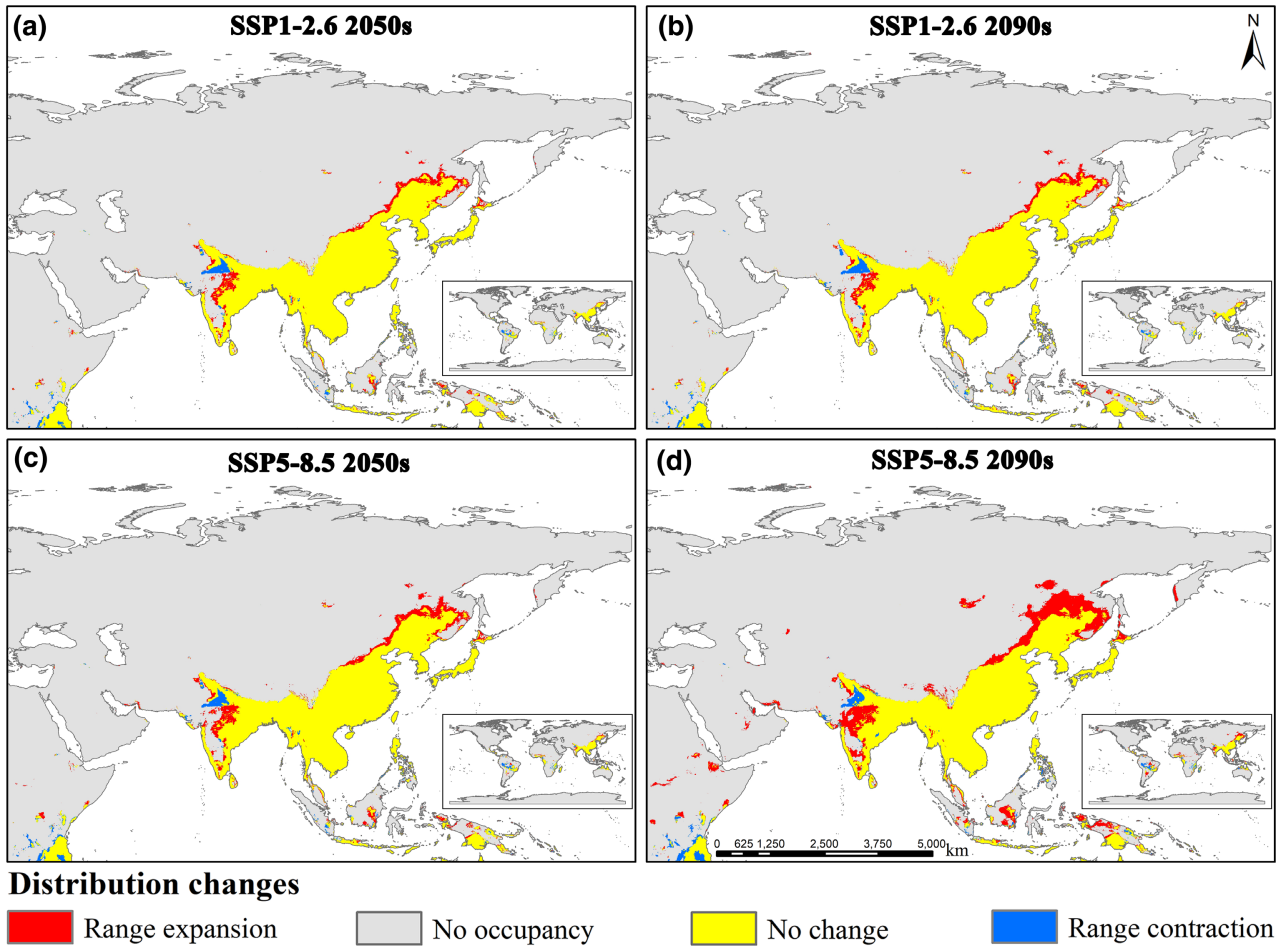
Changes in spatiotemporal distribution for *Limassolla* by ensemble model based on the binary distribution under future different climate scenarios in comparison with the current binary distribution. (a) SSP1‐2.62050s, (b) SSP1‐2.62090s, (c) SSP5‐8.52050s, (d) SSP5‐8.52090s.

Since the predictive results of the ensemble model are more accurate and robust than those of individual models (Araujo & New, 2007; Hao et al., 2019; Naimi & Araújo, 2016), the potential spatiotemporal distribution and centroid changes in this study are based on the ensemble model. Under different future climate scenarios, the change in suitable habitat area for *Limassolla* varies, but the overall range of change is generally consistent. Particularly, the stable regions (No change) remain largely unchanged, meaning that the areas currently suitable for *Limassolla* will likely remain suitable in the future. In the SSP1‐2.6 climate scenario, the expansion of suitable habitat area in the 2050s is estimated to be 92.28 × 10^4^ km^2^, and in the 2090s, it is projected to be 91.71 × 10^4^ km^2^. The expansion mainly occurs in the northern and northeastern China, as well as in neighboring regions of Russia, central India, Indonesia, and Hokkaido in Japan. On the other hand, the contraction of suitable habitat area in the 2050s is estimated to be 23.83 × 10^4^ km^2^, and it is projected to be 26.54 × 10^4^ km^2^ in the 2090s. The contraction primarily occurs in Punjab in Pakistan, the northwestern region of India, Magwe and Bago in Myanmar, Yunnan in China, Sumatra and Kalimantan in Indonesia, and the Philippines. In the SSP5‐8.5 climate scenario in the 2050s, the contraction area is 25.40 × 10^4^ km^2^, while suitable regions for the survival of *Limassolla* significantly expand, with a Asian expansion area of 118.56 × 10^4^ km^2^ (Figure 5, Table S5). The above results support our hypothesis 3.

**FIGURE 6:**
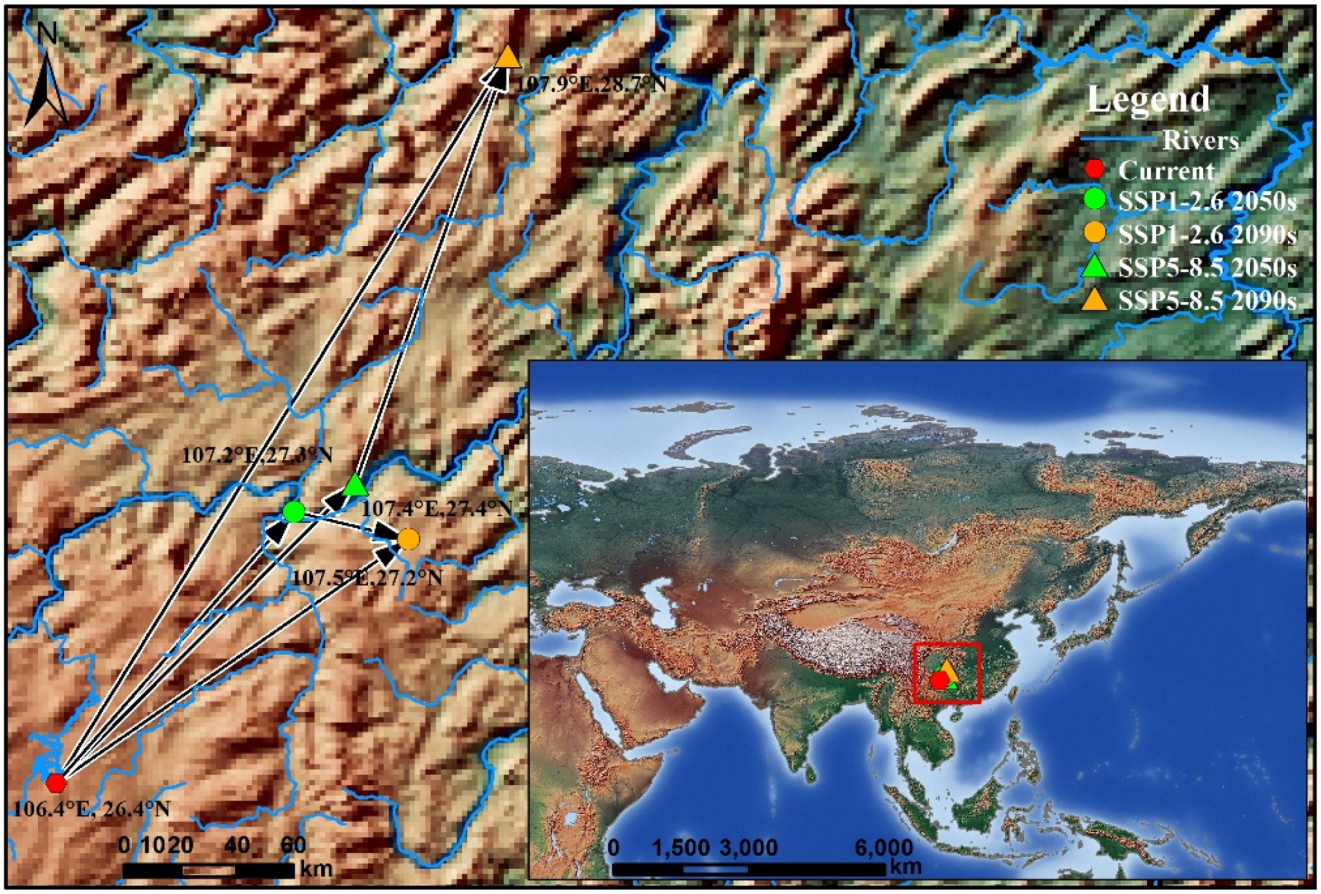
Centroid change in the potential Asian distribution of *Limassolla* under different climate scenarios by ensemble model. The square red box in the lower right corner represents the general distribution position of the centroid based on EM. The arrow represents the direction and magnitude of the distributional core shift over time.

The potential Asian distribution cores (centroid) of *Limassolla* in current and future climate scenarios are generally located in Guizhou, China. Currently, the centroid is situated at 106.43° E, 26.41° N. Under the SSP1‐2.6 climate scenario, the centroid shifts to 107.18° E, 27.27° N in the 2050s and further migrates to 107.55° E, 27.18° N in the 2090s. The impact on the distribution centroid of *Limassolla* is more pronounced under the SSP5‐8.5 climate scenario, with larger shifted distances. In the 2050s, the centroid is located at 107.38° E, 27.36° N, and in the 2090s, it shifts to 107.86° E, 28.72° N (Figure 6). It is evident that with climate change, the Asian distribution centroid of *Limassolla* tends to shift toward higher latitudes in the northeast direction, which supports our hypothesis 4.

## 4 DISCUSSION

### 4.1 Comparative analysis of single models and ensemble model

In this study, Maxlike, GLM, GAM, RF, and Maxent, together with their ensemble models, were used to predict the potential distribution of the *Limassolla*. While the predictions of the five individual models were generally consistent, differences still existed due to the different algorithms of each model, a common phenomenon in related studies (Araújo & Guisan, 2006; Elith et al., 2006; Hao et al., 2019; Liu et al., 2022; Pearson et al., 2006; Shabani et al., 2016). Maxlike is a formal likelihood model that estimates the probability of species occurrence and prevalence (Royle et al. 2012). The research findings confirmed that MaxLike accurately calculates the absolute probability of occurrence, whereas Maxent cannot (Merow & Silander, 2014). Maxent is a maximum entropy model that can generate accurate predictions even with limited species distribution data and is currently widely used and known for its effectiveness as an ecological niche model (Phillips et al. 2006). GLM is a conventional regression algorithm that permits response variables to display non‐normal error distributions, and is common algorithm for ENMs (Guo et al., 2019). GAM is a semi‐parametric extension of GLM, it is designed to handle species distribution responses and explanatory environmental variables with highly nonlinear and nonmonotonic relationships (Guo et al., 2019). The RF uses multiple decision trees and predicts according to the category with the highest vote rate and has been successfully used to predict the range of species distribution (Liu et al., 2022). Therefore, to address the variations caused by different modeling algorithms, we simultaneously employed an ensemble model combining these five models. This ensemble model reduces modeling uncertainties and provides more objective results (Elith & Leathwick, 2009; Hao et al., 2020; Srinivasulu et al., 2024). It combines the best‐performing models with the highest accuracy to achieve optimal results (Naimi & Araújo, 2016).

### 4.2 Main bioclimatic variables affecting the distribution of *Limassolla*

Due to the fact that different taxa may be influenced by different key environmental factors under climate change, for example, *Riptortus linearis* (Insecta: Hemiptera: Heteroptera: Alydidae) distributed in East Asia is most influenced by precipitation of wettest month (BIO13) (Fu et al., 2024), while *Cervus nippon* (Mammalia: Artiodactyla: Cervidae) distributed in East Asia is most influenced by annual precipitation (BIO12) (Luo et al., 2024), and *Callipogon relictus* (Coleoptera: Cerambycidae) distributed in Northeast Asia is most influenced by annual mean temperature (BIO1) (Kuprin et al., 2024). Therefore, understanding which environmental variables are most important is crucial for successfully predicting species' responses to climate change (Kellermann & Van Heerwaarden, 2019).

In this study, despite differences in the ranking of important variables determined by Maxlike, GLM, GAM, RF, and Maxent models, all variables can significantly explain the potential distribution of the *Limassolla*. Each model showed that precipitation of warmest quarter (BIO18) has the greatest impact on the distribution of *Limassolla*, rather than a bioclimatic variable that only includes temperature or precipitation, indicating that BIO18 contains more useful information for understanding the ecological niche of this taxon compared with other variables, which also supports our hypothesis 1. This result is completely consistent with the occurrence pattern of leafhoppers. Leafhoppers mostly occur in July and August of summer (Yang et al., 2022; Zhen‐Qiang et al., 2015), but it is difficult to collect leafhoppers in the field after rainfall or in excessively humid environments. This suggests that although summer is a peak period for leafhopper occurrence, precipitation during this season has a significant impact on leafhoppers' occurrence. Therefore, BIO18 is important for the occurrence of this insect group. On the contrary, all models indicated that precipitation of coldest quarter (BIO19) has the smallest impact on the distribution of this taxon. This is because leafhoppers are generally inactive during the coldest winter months and mostly overwinter as eggs (Yuan, 2020). Therefore, the precipitation during the coldest quarter has little effect on leafhoppers.

In addition, this population is almost distributed in tropical rainforest and subtropical evergreen broad‐leaved forest zones (Figures 3 and 4, Figure S3), where ample water and heat conditions indicate that this population is greatly influenced by precipitation and temperature. Numerous studies have also shown that precipitation and temperature are two of the most important factors affecting insect diversity and distribution (Haubrock et al., 2023; Lira et al., 2021; Liu et al., 2022). For example, research has shown that precipitation and temperature can affect the density of leafhopper populations (Scott et al., 2021), and temperature and precipitation days significantly impact the species diversity of typhlocybine leafhoppers (Wang et al., 2023).

### 4.3 Impacts of climate change on potential distribution of the *Limassolla*

ENMs has been proved by many studies to be a powerful technology for assessing the impact of climate change on insects, including leafhoppers (Aidoo et al., 2022, 2023; Azrag et al., 2022; Faleiro et al., 2018; Freer et al., 2022; Jin et al., 2022; Müller et al., 2019; Tepa‐Yotto et al., 2021). Correlational studies linking distributional shifts to environmental change provide evidence for adaptive responses to climate change of *Limassolla* species, with many species demonstrating the ability to adapt to warming through latitudinal shifts (Chen et al., 2011; Kerr et al. 2015; Konvicka et al., 2003; Lenoir & Svenning, 2015; Parmesan & Yohe, 2003; Thomas et al., 2001). In response to climate change, numerous species are undergoing poleward range expansions while exhibiting stability in range edges closer to the equator (Bebber et al., 2013; Frainer et al., 2017; Kerr et al. 2015). In the current CMIP6 model runs, the SSP1‐2.6 scenario projects an average warming of 2.0°C, while the SSP5‐8.5 scenario predicts an average warming of 5.0°C between 1880–1900 and 2090–2100. In this study, regardless of the SSP1‐2.6 sustainable development climate scenario or the SSP5‐8.5 pessimistic climate scenario, based on EM, the Asian distribution core of *Limassolla* is projected to shift toward higher latitudes in the northeast direction. The direction of shift is from the current Pingba District in Anshun City, Guizhou province, China to the Weng'an County in the Qiannan Buyi and Miao Autonomous Prefecture in Guizhou, China (SSP1‐2.6 2050s, SSP1‐2.6 2090s, and SSP5‐5.8 2050s), Wuchuan Gelao and Miao Autonomous County, Zunyi City, Guizhou, China (SSP5‐5.8 2090s). In the SSP5‐8.5 climate scenario, the distribution core of *Limassolla* moves farther (Figure 6), indicating a stronger impact on its distribution under a high‐emission development pathway. Therefore, we need to take the path of sustainable development with low emissions.

Under climate change, not all leafhopper's suitable habitats exhibit the same trend as *Limassolla* leafhoppers. For instance, it is predicted that by 2050s, even under a moderate climate change scenario, the habitat area of the *Subpsaltria yangi* (Hemiptera: Cicadidae), will significantly decrease (Zhen‐Peng et al., 2019). Unlike it, in this study, it is predicted that by 2050, regardless of the moderate SSP1‐2.6 or the extreme emissions SSP5‐8.5 climate scenarios, the suitable habitat area of *Limassolla* will increase (Table S4). For the tea green leafhoppers, researchers indicated that under both SSP2‐4.5 and SSP5‐8.5 scenarios in 2050s and 2070s, the suitable habitat area of the tea green leafhoppers have increased compared to the current situation, and the increase is the largest under the SSP5‐8.5 climate scenario (Jiang et al., 2022). In this study, the *Limassolla* leafhoppers also see the largest increase under the SSP5‐8.5 climate scenario (Tables S4 and S5), and the suitable distribution area of *Limassolla* has increased under different climate scenarios during the nearer future and longer term periods. This is because climate change generates novel ecological niches, enabling insects to establish, expand, and shift their distribution to previously unoccupied geographic regions (Skendžić et al., 2021). However, for species with very weak dispersal capacity, this will undoubtedly lead to the extinction of the species, suggesting that future conservation status should be strengthened due to climate change.

Furthermore, combining Sections 3.3, 3.5, although *Limassolla*'s primary distribution under future climate change remains largely consistent with the current distribution, there are both expansion and contraction areas. The presence of contraction areas implies habitat loss, which supports our hypothesis 2. The contraction of suitable habitat areas of *Limassolla* mostly occurs in tropical regions, while expansion occurs primarily in temperate regions (Figure 6). This is due to future climate warming will lead to higher frequencies of high temperatures, and it is expected that the average adaptability and population growth rate of insects may rise in temperate regions, while it will decline in tropical regions (Deutsch et al., 2008, 2018; Kingsolver et al., 2011). Recent reports indicate a decline in global insect populations (Wagner, 2020). With global warming, a significant number of species are expected to go extinct. The extent of future climate change is evidently a key factor in predicting the risk of extinction (Hance et al., 2007; Lambers, 2015). Therefore, it is necessary to assess the distribution of the *Limassolla* under different future climate scenarios. At the same time, it is important to focus on achieving harmony between humans and nature. To address global climate warming, all of humanity should unite to adopt low‐carbon lifestyles to mitigate the pace of global warming. Additionally, the establishment of ecological conservation areas to protect biodiversity is crucial.

### 4.4 Implications for biological and environmental conservation

Global biodiversity is facing a threat due to habitat loss and climate change‐induced alterations (Zhu et al., 2022), which impact biodiversity at various levels, from individual organisms to entire ecosystems (Peñuelas et al., 2013). Therefore, in the context of global climate change, there is an urgent need for environmental and biodiversity conservation efforts, with the crucial question being where to implement these conservation measures. In this study, we analyze the distribution changes and potential shift directions of the *Limassolla* leafhoppers under climate change, aiming to address the question of “where conservation measures are needed in the face of global climate change?” Compared with other more migratory insects, the close association of leafhoppers with their host plants endows them with high spatial resolution (Nickel & Achtziger, 2005). Their limited dispersal ability allows them to actively respond to changes in the climate and environment, making them ideal subjects for biogeographical studies (Hill et al., 2009; Wang et al., 2012) and models for identifying biodiversity hotspots (Liu et al., 2022). Moreover, as one kind of ideal bioindicators, leafhoppers have been used to monitor and evaluate ecological restoration in karst rocky desertification control areas in southwest China, with their distribution patterns serving as indicators for environmental monitoring (Chen et al., 2022; Zhen‐Qiang et al., 2015). Numerous scholars have supported the use of leafhoppers as bioindicators of disturbance intensity and habitat conditions, making them valuable tools for flora and fauna conservation (Borchard & Fartmann, 2014; Hollier et al., 2005). Therefore, we utilized the characteristic of leafhoppers as bioindicators sensitive to climate and environmental changes to provide invaluable insights into the conservation of both the environment and organisms in this study.

The areas with high suitability identified as potential biodiversity hotspots will provide valuable guidance for determining priority protected areas and implementing protective measures in the future. The 11 biodiversity hotspots identified in this study are located in mountainous areas (Figure 3f). These mountainous areas, known for their unique and diverse natural environments, often harbor rich biodiversity, including various endangered and endemic species (Fan et al., 2024; Li et al., 2021). Previous researchers have used the high suitability areas of neotenic net‐winged beetle to identify biodiversity hotspots in China, such as the Hengduan Mountains, Daba Shan, Wu‐chih Mountains, and Central Range (Liu et al., 2022). Additionally, this study also identifies the Ta‐pieh Mountains and Mount Tai as biodiversity hotspots (Figure 3). Comparative analysis of Figures 3 and 5 reveals that future contraction areas primarily originate from the current low suitability zones. Therefore, it is imperative to establish ecological conservation areas within our identified contraction zones to safeguard biodiversity, and the low suitability areas should not be neglected to be protected. Thus, our research findings contribute to the implementation of effective management policies for *Limassolla*, as well as other organisms and the overall ecosystem, in pursuit of sustainable development.

Although the predictive performance of all models is outstanding, there are still limitations. Species distribution patterns are influenced not only by temperature and precipitation but also by factors such as adaptability, competitive ability with other species in the same habitat, wind direction and strength, and human activities (Saha et al., 2021; Santana et al., 2019). With the continuous development of research techniques, we will consider adding more factors as supplementary data in the model to make its prediction results more accurate. Despite the limitations, the models still have broad applicability in current fields such as ecology, biogeography, and biodiversity conservation (Werkowska et al., 2017). The research subjects can be terrestrial plants and animals (Crespo‐Pérez et al., 2023; Higgins et al., 2020), marine organisms (Thorson & Barnett, 2017; Zhang et al. 2022c), microorganisms (Mod et al., 2020; Pajunen et al., 2016), parasitic diseases (Hu et al., 2020; Mod et al., 2020; Pajunen et al., 2016), among others. In the future, interdisciplinary cooperation can be strengthened to incorporate knowledge from multiple fields such as social sciences into the models.

## 5 CONCLUSIONS

This study conducted a comprehensive analysis of the distribution of *Limassolla* based on different Shared Socio‐economic Pathways and climate scenarios, incorporating theories and methods from insect ecology, environmental ecology, and biogeography. Our research results supported the four hypotheses we proposed. The research results indicated that *Limassolla* is mainly distributed in East Asia, South Asia, and Southeast Asia. With climate change, the distribution range of *Limassolla* will expand in some regions while also contract in others, with the distribution core shifting toward higher latitudes in the northeast direction. Eleven regions have been identified as potential biodiversity hotspots. The contribution of precipitation of warmest quarter to the models is the highest and has the greatest impact on the distribution of *Limassolla*. Although the total suitable habitat area for *Limassolla* leafhoppers is expected to increase in the future, considering the simultaneous loss of habitats, the weak dispersal ability of species, and the instability of climate change, it is still necessary to be vigilant and strengthen the conservation status in the future. Therefore, it is necessary to establish ecological reserves in contraction areas that we have identified to protect biodiversity. The research results provided previously unpublished information on the ecological niche of *Limassolla*, which has valuable implications for the development of effective environmental and biodiversity conservation measures, as well as the discovery of new species.

## AUTHOR CONTRIBUTIONS

**Weiwei Ran:** Conceptualization (equal); data curation (equal); methodology (equal); methodology (equal); software (equal); software (equal); validation (equal); validation (equal); visualization (equal); visualization (equal); writing – original draft (equal); writing – original draft (equal). **Jiajia Chen:** Formal analysis (equal); visualization (equal). **Yuanqi Zhao:** Writing – review and editing (equal). **Ni Zhang:** Writing – review and editing (equal). **Guimei Luo:** Data curation (equal). **Zhibing Zhao:** Investigation (equal). **Yuehua Song:** Funding acquisition (equal); resources (equal); supervision (equal); writing – review and editing (equal).

## FUNDING INFORMATION

This study was funded by the National Natural Science Foundation of China (32260120), the Natural Science Foundation of Guizhou Province (Qiankehejichu‐ZK [2023] General 257), the Training Program for High‐level Innovative Talents of Guizhou Province (Qiankehepingtairencai‐GCC [2023]032), and the Science and Technology Innovation Talent Team Building Project of Guizhou Province (Qiankehepingtairencai‐CXTD [2023]010).

## CONFLICT OF INTEREST STATEMENT

The authors have no relevant financial or nonfinancial interests to disclose.

## Supporting information


Appendix S1.

## Data Availability

The data that support the findings of this study are available in the supporting information of this article.
