# Supplementary material for: Global climate change‐driven impacts on the Asian distribution of Limassolla leafhoppers, with implications for biological and environmental conservation
Source: Ecol Evol. 2024 Jul 18;14(7):e70003. doi: 10.1002/ece3.70003 (PMC11257772; doi:10.1002/ece3.70003)
Supplement: Supplementary file 1 — Appendix S1. [file ECE3-14-e70003-s001.docx]

## *Ecology and Evolution*

## Supporting Information

## Global climate change-driven impacts on the Asian distribution of Limassolla leafhoppers, with implications for biological and environmental conservation

Weiwei Ran^1,2^ **·** Jiajia Chen^1,2^ **·** Yuanqi Zhao^1,2^ **·** Ni Zhang^1,2^ **·** Guimei Luo^1,2^ **·** Zhibing Zhao^1,2,3^ **·** Yuehua Song^1,2*^

^1^ School of Karst Science, Guizhou Normal University, Guiyang 550001, China

^2^ State Engineering Technology Institute for Karst Desertification Control, Guiyang 550001, China

^3^ School of Food Science and Engineering, Guiyang University, Guiyang, Guizhou 550005, China

^*^ Correspondence: [songyuehua@163.com](mailto:songyuehua@163.com)

**Contents**

**Table S1** The occurrence records of *Limassolla* in modeling **(Page 2-8)**

**Fig. S1** Pearson correlation analysis among the nineteen bioclimatic variables **(Page 8)**

**Fig. S2** The mean accuracy assessment of the twelve models using different evaluation metrics **(Page 9)**

**Table S2** Nineteen bioclimatic variables used for modeling **(Page 9-10)**

**Table S3** The mean accuracy assessment of the five models using different evaluation metrics **(Page 10)**

**Table S4** Potential Asian suitable area for *Limassolla* using ensemble modeling under current and future climate scenarios (10^4^ km^2^) **(Page11)**

**Table S5** Asian spatiotemporal distribution change area of *Limassolla* by ensemble model based on the binary distribution under future different climate scenarios in comparison to the current binary distribution (10^4^ km^2^) **(Page 11)**

**Fig. S3** Habitat suitability of *Limassolla* under future SSP5-8.5 climate scenario using Maxlike, GLM, GAM, RF, Maxent, and EM **(Page 12)**

**Table S1** The occurrence records of *Limassolla* in modeling

| **Species** | **Number of species' occurrence records** | **Longitude** | **Latitude** | **Source** |
| --- | --- | --- | --- | --- |
| *Limassolla aureata* (Mahmood, 1967) | 1 | 95.955974 | 21.916221 | *ERYTHRONEURINI AND ZYGINELLINI FROM CHINA* |
| *Limassolla auriculata* Song & Li 2011 | 1 | 108.186282 | 26.39567 | *ERYTHRONEURINI AND ZYGINELLINI FROM CHINA* |
| *Limassolla bengalensis* (Ahmed, 1972) | 2 | 91.18331 | 23.453059 | Ahmed (1972) |
|  |  | 100.896103 | 22.087017 | *ERYTHRONEURINI AND ZYGINELLINI FROM CHINA* |
| *Limassolla bicruralis* Huang & Zhang, 2020 | 1 | 120.3014353 | 22.6272784 | Dworakowska (1972) |
| *Limassolla bielawskii* Dworakowska, 1969 | 3 | 114.75821 | 23.02253 | Dworakowska (1969) |
|  |  | 104.215069 | 31.741017 | Song & Li (2011) |
|  |  | 100.301282 | 21.938906 | *ERYTHRONEURINI AND ZYGINELLINI FROM CHINA* |
| *Limassolla dangjinensis* Oh et Jung, 2020 | 2 | 127.7599737 | 36.1014755 | Oh & Jung (2022) |
|  |  | 102.7097299 | 25.0452999 | Huang & Zhang (2020) |
| *Limassolla diospyri* Chou & Ma, 1981 | 6 | 108.275775 | 28.593899 | Song & Li (2011) |
|  |  | 107.886139 | 28.544033 | *ERYTHRONEURINI AND ZYGINELLINI FROM CHINA* |
|  |  | 108.830475 | 19.057516 | *ERYTHRONEURINI AND ZYGINELLINI FROM CHINA* |
|  |  | 108.875116 | 18.714954 | Huang & Zhang (2020) |
|  |  | 114.572121 | 24.668068 | *ERYTHRONEURINI AND ZYGINELLINI FROM CHINA* |
|  |  | 106.019655 | 32.680832 | *ERYTHRONEURINI AND ZYGINELLINI FROM CHINA* |
| *Limassolla discoloris* Zhang & Chou, 1988 | 2 | 108.792488 | 18.690415 | *ERYTHRONEURINI AND ZYGINELLINI FROM CHINA* |
|  |  | 108.20018 | 34.2603599 | Chou & Ma (1981) |
| *Limassolla discreta* Chou & Zhang, 1985 | 3 | 43.6854936 | -23.3516191 | Dworakowska (1997) |
|  |  | 106.671819 | 26.421877 | *ERYTHRONEURINI AND ZYGINELLINI FROM CHINA* |
|  |  | 108.07754 | 26.378443 | Song & Li (2011) |
| *Limassolla dispunctata* Chou & Ma, 1981 | 3 | 108.277199 | 14.058324 | Dworakowska (1969) |
|  |  | 109.830436 | 18.728495 | Song & Li (2011) |
|  |  | 110.1484195 | 34.0908202 | Chou & Ma (1981) |
| *Limassolla dostali* Dworakowska & Lauterer, 1975 | 6 | 96.173526 | 16.840939 | Dworakowska & Lauterer (1975) |
|  |  | -9.696645 | 9.945587 | *ERYTHRONEURINI AND ZYGINELLINI FROM CHINA* |
|  |  | 127.4361117 | 36.8553798 | Hossain et al. (2019) |
|  |  | 109.6975 | 18.7283333 | Huang & Zhang (2020) |
|  |  | 100.45223 | 21.9575299 | *ERYTHRONEURINI AND ZYGINELLINI FROM CHINA* |
|  |  | 113.26627 | 23.1317099 | *ERYTHRONEURINI AND ZYGINELLINI FROM CHINA* |
| *Limassolla dworakowskae* Chou & Ma, 1981 | 1 | 117.1571 | 37.3090699 | *ERYTHRONEURINI AND ZYGINELLINI FROM CHINA* |
| *Limassolla emmrichi* Dworakowska, 1972 | 3 | 138.252924 | 36.204824 | *ERYTHRONEURINI AND ZYGINELLINI FROM CHINA* |
|  |  | 112.847011 | 24.980018 | Zhang & Chou (1988) |
|  |  | 106.4140868 | 26.4776829 | Song & Li (2011) |
| *Limassolla erythromaculatus* (Ramakrishnan & Menon, 1972) | 2 | 77.1571443 | 28.6376724 | Ramakrishnan & Menon (1972) |
|  |  | 106.70722 | 26.5981999 | *ERYTHRONEURINI AND ZYGINELLINI FROM CHINA* |
| *Limassolla fasciata* Zhang & Chou, 1988 | 3 | 108.180608 | 28.651988 | *ERYTHRONEURINI AND ZYGINELLINI FROM CHINA* |
|  |  | 107.86971 | 25.9832599 | *ERYTHRONEURINI AND ZYGINELLINI FROM CHINA* |
|  |  | 116.45599 | 36.2893699 | *ERYTHRONEURINI AND ZYGINELLINI FROM CHINA* |
| *Limassolla forcipata* Song & Li, 2011 | 2 | 114.440702 | 24.577006 | *ERYTHRONEURINI AND ZYGINELLINI FROM CHINA* |
|  |  | 121.77314 | 37.2501699 | Huang & Zhang (2020) |
| *Limassolla galewskii* Dworakowska, 1969 | 3 | 147.736287 | -8.878839 | Dworakowska (1972) |
|  |  | 109.261896 | 19.134655 | Chou & Zhang (1986) |
|  |  | 109.171844 | 18.357288 | Chou & Zhang (1985) |
| *Limassolla georgei* Mathew & Ramakrishnan, 2002 | 2 | 76.5748309 | 9.8079628 | Mathew & Ramakrishnan (2002) |
|  |  | 112.9833341 | 28.1142216 | *ERYTHRONEURINI AND ZYGINELLINI FROM CHINA* |
| *Limassolla gratiosa* (Dworakowska, 1981) | 1 | 88.57741989 | 27.41642318 | Dworakowska (1994) |
| *Limassolla hebeiensis* Cai, Liang & Wang, 1992 | 8 | 76.3608343 | 10.5270382 | Mathew & Ramakrishnan (2002) |
|  |  | 126.7133045 | 36.8438455 | Oh & Jung (2020) |
|  |  | 103.84425 | 1.314 | Mahmood (1967) |
|  |  | 106.75171 | 25.4253599 | *ERYTHRONEURINI AND ZYGINELLINI FROM CHINA* |
|  |  | 104.07572 | 30.6508899 | *ERYTHRONEURINI AND ZYGINELLINI FROM CHINA* |
|  |  | 88.1364591 | 43.887985 | *ERYTHRONEURINI AND ZYGINELLINI FROM CHINA* |
|  |  | 118.76295 | 32.0607099 | Dworakowska (1969) |
|  |  | 120.1536 | 30.2655499 | *ERYTHRONEURINI AND ZYGINELLINI FROM CHINA* |
| *Limassolla ishiharai* Dworakowska, 1972 | 14 | 128.003245 | 37.097721 | Hossain et al (2019) |
|  |  | 139.6503106 | 35.6761919 | Dworakowska (1972) |
|  |  | 127.8653897 | 36.2684433 | Oh et Jung (2021) |
|  |  | 126.6393969 | 36.4500512 | Oh & Jung (2020) |
|  |  | 127.263403 | 37.0826802 | Oh & Jung (2021) |
|  |  | 127.7202079 | 35.8369808 | Oh & Jung (2020) |
|  |  | 26.258017 | -9.226924 | Dworakowska (1981) |
|  |  | 110.202004 | 18.75622 | Chou & Zhang (1988) |
|  |  | 110.48579 | 33.4619599 | Chou & Ma (1981) |
|  |  | 121.0342254 | 24.06726621 | Chiang et al. (1988) |
|  |  | 99.16181 | 25.1120499 | Chou & Ma (1981) |
|  |  | 109.5425 | 19.0358333 | Huang & Zhang (2020) |
|  |  | 108.95424 | 34.2648599 | *ERYTHRONEURINI AND ZYGINELLINI FROM CHINA* |
|  |  | 102.52744 | 24.3472599 | Zhang & Xiao (2000) |
| *Limassolla kakii* Chou & Ma, 1981 | 1 | 109.494414 | 19.507339 | Chou & Zhang (1987) |
| *Limassolla karachiensis* (Ahmed & Jabbar, 1971） | 1 | 128.114498 | 37.652614 | Hossain et al (2020) |
| *Limassolla knighti* Dworakowska, 1972 | 1 | 128.679577 | 37.361782 | Hossain et al (2019) |
| *Limassolla koreana* Hossain, Kwon, Suh & Kwon, 2019 | 5 | 126.9931482 | 36.9768754 | Hossain et al. (2019) |
|  |  | 127.0262077 | 34.6793275 | Hossain et al. (2019) |
|  |  | 127.9745517 | 36.3380774 | Oh et Jung (2022) |
|  |  | 108.32754 | 22.8152099 | *ERYTHRONEURINI AND ZYGINELLINI FROM CHINA* |
|  |  | 113.75322 | 34.7657099 | *ERYTHRONEURINI AND ZYGINELLINI FROM CHINA* |
| *Limassolla krasna* *insulana* (Dworakowska, 1981) | 3 | -5.54708 | 7.539989 | Dworakowska (1981) |
|  |  | 126.632629 | 36.672331 | Hossain et al (2019) |
|  |  | 108.6902778 | 27.9202778 | Huang & Zhang (2020) |
| *Limassolla kunyica* Huang & Zhang, 2020 | 1 | 104.8955299 | 25.0919199 | *ERYTHRONEURINI AND ZYGINELLINI FROM CHINA* |
| *Limassolla lanyua* Chiang, Hsu & Knight, 1989 | 1 | 113.04352 | 25.79628 | Zhang & Chou (1988) |
| *Limassolla lingchuanensis* Chou & Zhang, 1985 | 5 | 110.34863 | 20.0199699 | *ERYTHRONEURINI AND ZYGINELLINI FROM CHINA* |
|  |  | 29.7136456 | 11.0105443 | Dworakowska (1972) |
|  |  | 105.9582372 | 27.1981851 | Song & Li (2011) |
|  |  | 114.50443 | 37.0705499 | Cai et al. (1992) |
|  |  | 108.35342 | 21.7691299 | Huang & Zhang (2020) |
| *Limassolla malgaska* Dworakowska, 1997 | 1 | 8.389935 | 9.602702 | Dworakowska (1981) |
| *Limassolla multimacula* Chiang, Lee & Knight, 1988 | 2 | 67.0011364 | 24.8607343 | Sohi & Pathania (2012) |
|  |  | 111.615769 | 33.785698 | *ERYTHRONEURINI AND ZYGINELLINI FROM CHINA* |
| *Limassolla multipunctata* (Matsumura, 1920) | 3 | 103.8437852 | 22.3363608 | Dworakowska (1977) |
|  |  | 116.74503 | 32.1533999 | Cai et al. (1993) |
|  |  | 105.326397 | 27.306657 | *ERYTHRONEURINI AND ZYGINELLINI FROM CHINA* |
| *Limassolla nigropunctata* Huang & Zhang, 2020 | 1 | 108.45295 | 33.43243 | Zhang & Chou (1988) |
| *Limassolla pistaciae* (Linnavuori, 1962) | 1 | 8.8928048 | 9.9052928 | Dworakowska (1981) |
| *Limassolla qianfoensis* Song & Li, 2011 | 1 | 112.1666667 | 35.2 | *ERYTHRONEURINI AND ZYGINELLINI FROM CHINA* |
| *Limassolla rubrolimbata* Zhang & Chou, 1988 | 3 | 108.492756 | 28.568769 | *ERYTHRONEURINI AND ZYGINELLINI FROM CHINA* |
|  |  | 104.47158 | 25.7099299 | *ERYTHRONEURINI AND ZYGINELLINI FROM CHINA* |
|  |  | 109.532589 | 18.936946 | Huang & Zhang (2020) |
| *Limassolla rutila* Song & Li, 2011 | 4 | 121.076136 | 24.233586 | Chiang et al. (1988) |
|  |  | 27.5026174 | -11.6876026 | Dworakowska (1981) |
|  |  | 119.2965899 | 26.0998199 | *ERYTHRONEURINI AND ZYGINELLINI FROM CHINA* |
|  |  | 109.3652 | 32.8341699 | Zhang & Chou (1988) |
| *Limassolla schmitzi* (Dworakowska, 1981) | 1 | 128.593586 | 38.20097 | Oh & Jung (2020) |
| *Limassolla spinulata* Huang & Zhang, 2020 | 3 | 107.51867 | 26.2597899 | *ERYTHRONEURINI AND ZYGINELLINI FROM CHINA* |
|  |  | 107.886969 | 25.411157 | *ERYTHRONEURINI AND ZYGINELLINI FROM CHINA* |
|  |  | 109.15258 | 33.4233699 | Zhang & Chou (1988) |
| *Limassolla uncata* Huang & Zhang, 2020 | 1 | 108.86842 | 34.04742 | *ERYTHRONEURINI AND ZYGINELLINI FROM CHINA* |
| *Limassolla unica* Zhang & Xiao, 2000 | 1 | 121.548418 | 22.0435616 | Chiang et al. (1989) |
| *Limassolla yingjianga* Song & Li, 2011 | 1 | 109.007286 | 33.981478 | *ERYTHRONEURINI AND ZYGINELLINI FROM CHINA* |
| *Limassolla zelta* Dworakowska, 1977 | 1 | 88.2400333 | 27.3197716 | Dworakowska (1994) |
| *Limassolla zhangi* Dworakowska, 1994 | 3 | 88.6138113 | 27.3313512 | Dworakowska (1994) |
|  |  | 45.55 | -24.316667 | Dworakowska (1981) |
|  |  | 97.65621 | 24.61217 | *ERYTHRONEURINI AND ZYGINELLINI FROM CHINA* |


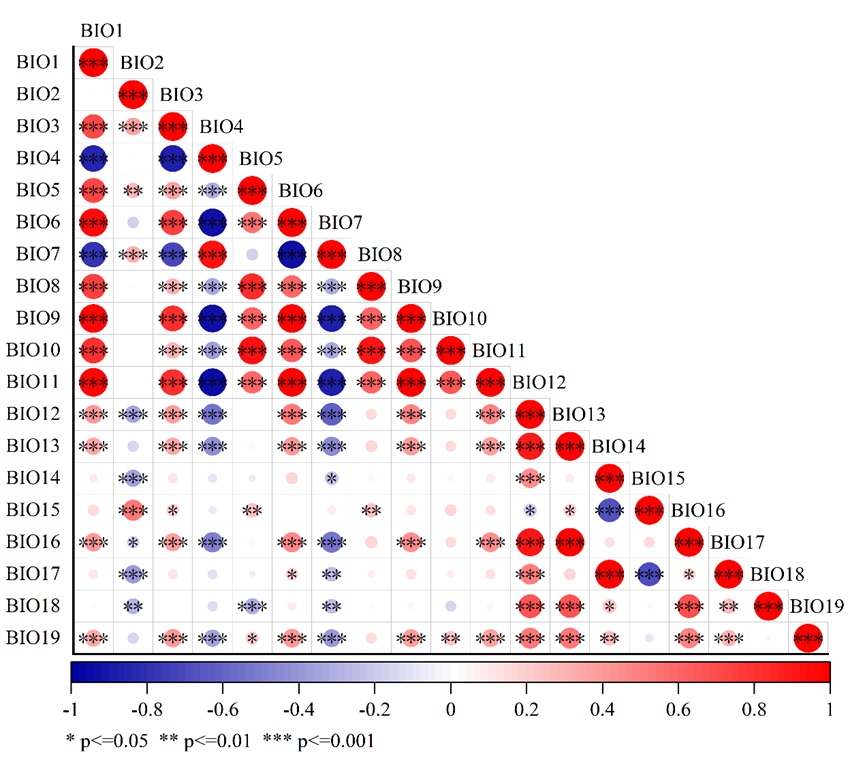


**Fig. S1** Pearson correlation analysis among the nineteen bioclimatic variables. Warm colors represent positive correlations between two bioclimatic variables, with darker shades and larger circles indicating stronger positive correlations; while cool colors represent negative correlations between two bioclimatic variables, with darker shades and larger circles indicating stronger negative correlations.


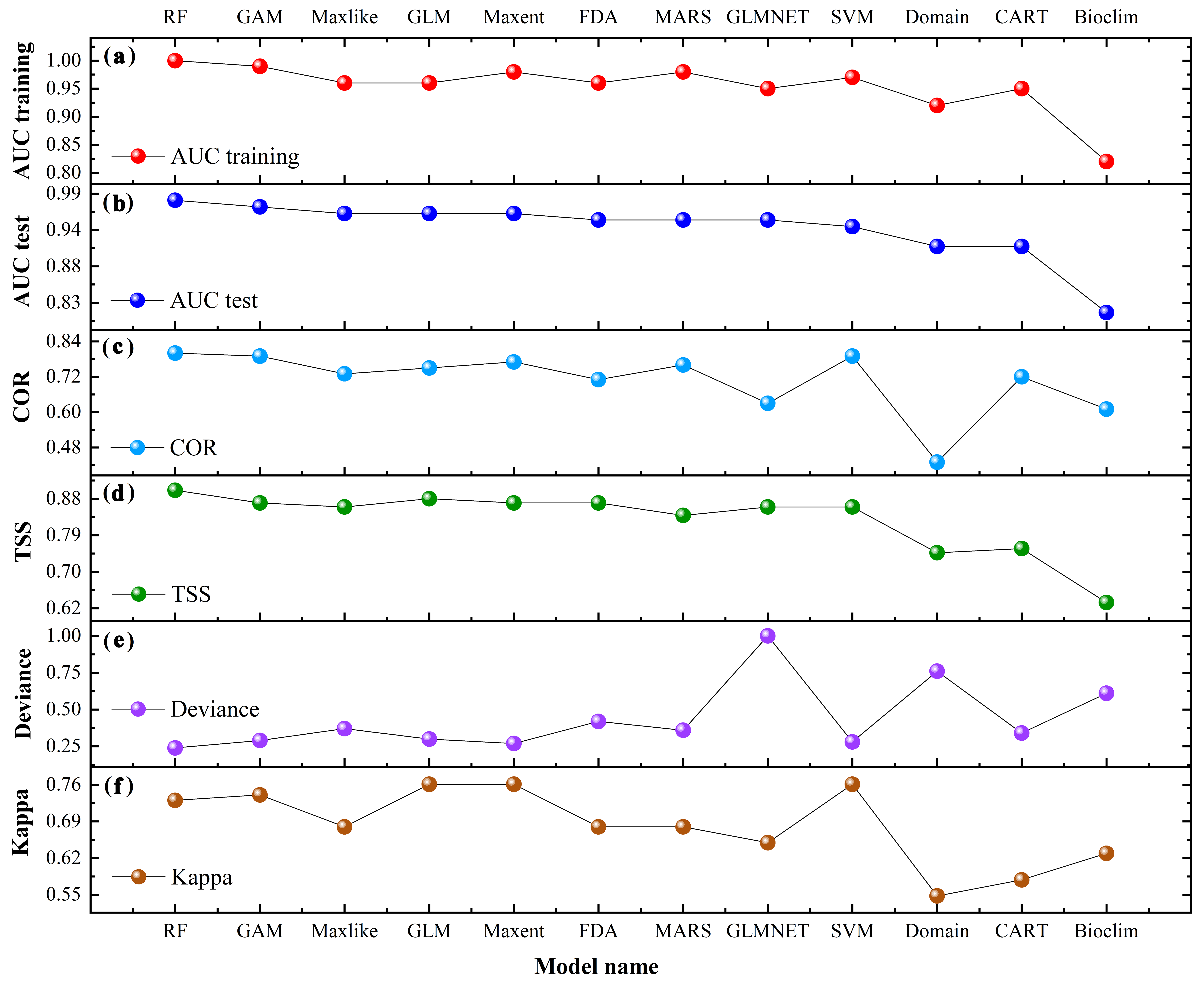


**Fig. S2** The mean accuracy assessment of the twelve models using different evaluation metrics, (a) area under the curve (AUC) training, (b) AUC test, (c) correlation (COR), (d) true skill statistic (TSS), (e) Deviance, and (f) Kappa based a 10-fold cross-validation approach

**Table S2** Nineteen bioclimatic variables used for modeling

| Variables | Description | Units | Percent contribution |
| --- | --- | --- | --- |
| BIO1 | Annual Mean Temperature | ℃ | 0.55 |
| BIO2^*^ | Mean Diurnal Range (Mean of monthly (max temp - min temp)) | ℃ | 3.49 |
| BIO3 | Isothermality (BIO2/BIO7) (×100) | – | 4.64 |
| BIO4^*^ | Temperature Seasonality (standard deviation ×100) | – | 9.62 |
| BIO5 | Max Temperature of Warmest Month | ℃ | 0.15 |
| BIO6 | Min Temperature of Coldest Month | ℃ | 1.87 |
| BIO7 | Temperature Annual Range (BIO5-BIO6) | ℃ | 2.30 |
| BIO8 | Mean Temperature of Wettest Quarter | ℃ | 0.75 |
| BIO9 | Mean Temperature of Driest Quarter | ℃ | 0.43 |
| BIO10^*^ | Mean Temperature of Warmest Quarter | ℃ | 1.12 |
| BIO11 | Mean Temperature of Coldest Quarter | ℃ | 1.33 |
| BIO12 | Annual Precipitation | mm | 0.22 |
| BIO13^*^ | Precipitation of Wettest Month | mm | 21.77 |
| BIO14 | Precipitation of Driest Month | mm | 0.30 |
| BIO15^*^ | Precipitation Seasonality (Coefficient of Variation) | – | 8.43 |
| BIO16 | Precipitation of Wettest Quarter | mm | 0.71 |
| BIO17^*^ | Precipitation of Driest Quarter | mm | 0.36 |
| BIO18^*^ | Precipitation of Warmest Quarter | mm | 40.38 |
| BIO19^*^ | Precipitation of Coldest Quarter | mm | 0.55 |

Note: ^*^Means the bioclimatic variables used in final models. The percent contribution was only obtained based on MaxEnt model

**Table S3** The mean accuracy assessment of the five models using different evaluation metrics, area under the curve (AUC), correlation (COR), true skill statistic (TSS), deviance, and the Kappa based a 10-fold cross-validation approach

| **Model name** | **AUC training** | **AUC test** | **COR** | **TSS** | **Deviance** | **Kappa** |
| --- | --- | --- | --- | --- | --- | --- |
| Maxlike | 0.96 | 0.96 | 0.73 | 0.86 | 0.37 | 0.68 |
| Generalized linear model (GLM) | 0.96 | 0.96 | 0.75 | 0.88 | 0.3 | 0.76 |
| Generalized additive model (GAM) | 0.99 | 0.97 | 0.79 | 0.87 | 0.29 | 0.74 |
| Random forest (RF) | 1 | 0.98 | 0.8 | 0.9 | 0.24 | 0.72 |
| Maximum entropy (Maxent) | 0.98 | 0.96 | 0.77 | 0.87 | 0.27 | 0.76 |

**Table S4** Potential Asian suitable area for *Limassolla* using ensemble modeling under current and future climate scenarios (10^4^ km^2^)

| Suitability class | Current | SSP1-2.6 2050s | SSP1-2.6 2090s | SSP5-8.5 2050s | SSP5-8.5 2090s |
| --- | --- | --- | --- | --- | --- |
| low suitability  (0.2-0.4) | 245.76 | 261.74 | 267.99 | 263.33 | 307.09 |
| moderate suitability  (0.4-0.6) | 163.43 | 187.88 | 189.83 | 198.65 | 251.22 |
| optimum suitability  (0.6-0.8) | 159.42 | 177.43 | 172.54 | 178.02 | 198.53 |
| high suitability  (0.8-1.0) | 153.39 | 152.24 | 147.21 | 160.71 | 154.83 |
| suitable habitat  (0.2-1.0) | 722.00 | 779.29  (57.29) | 777.57  (55.57) | 800.70  (78.71) | 911.67  (189.67) |

The values in parentheses represent the increase in the suitable area compared to the current climate scenario

**Table S5** Asian spatiotemporal distribution change area of *Limassolla* by ensemble model based on the binary distribution under future different climate scenarios in comparison to the current binary distribution (10^4^ km^2^)

| Climate scenarios | Range expansion | No occupancy (absence in both) | No change (presence in both) | Range contraction |
| --- | --- | --- | --- | --- |
| SSP1-2.6 2050s | 92.28 | 3421.18 | 920.61 | 23.83 |
| SSP1-2.6 2090s | 91.71 | 3421.74 | 917.91 | 26.54 |
| SSP5-8.5 2050s | 118.56 | 3394.90 | 919.05 | 25.40 |
| SSP5-8.5 2090s | 243.60 | 3269.85 | 918.64 | 25.81 |


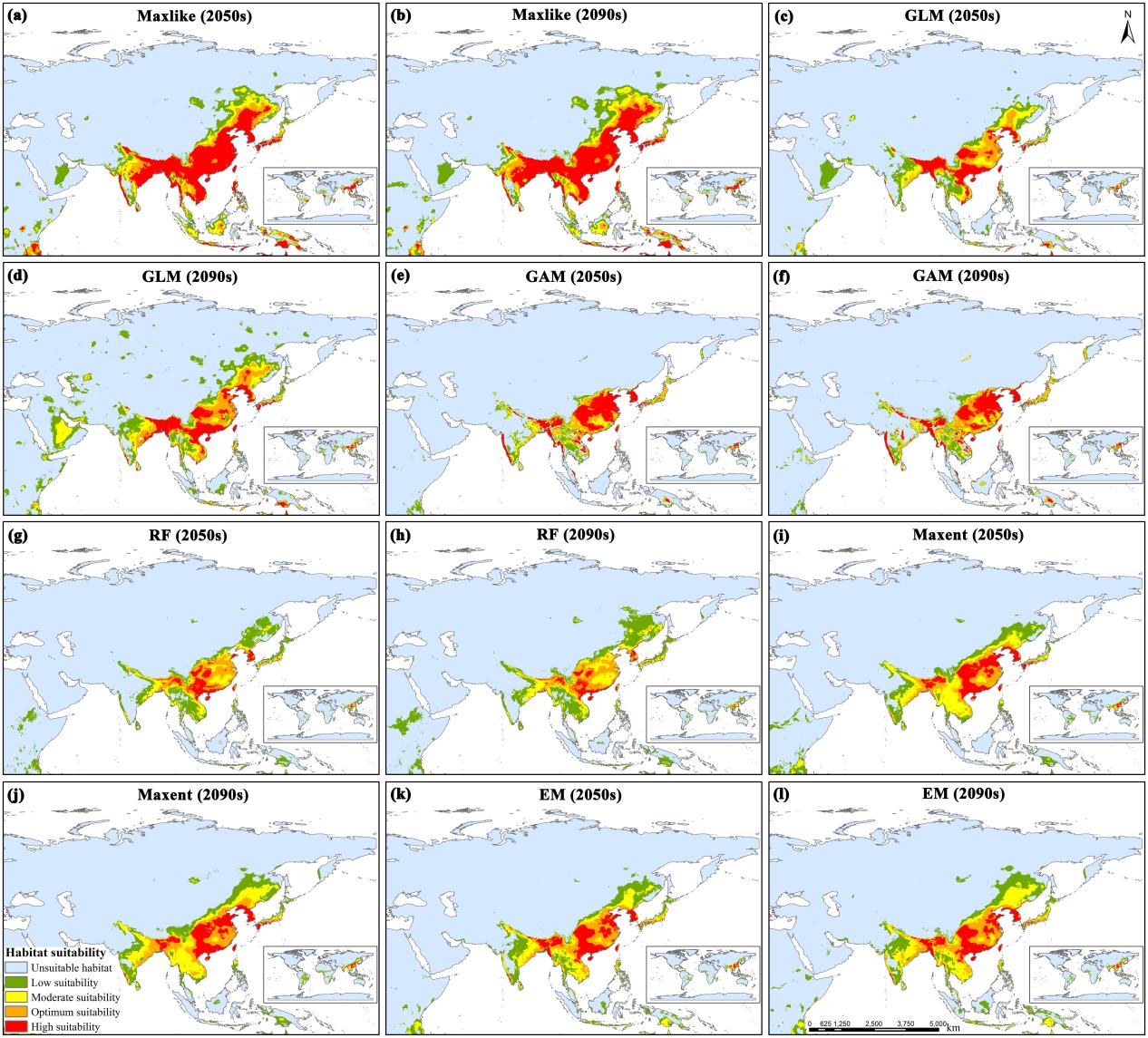


**Fig. S3** Habitat suitability of *Limassolla* under future SSP5-8.5 climate scenario using Maxlike, GLM, GAM, RF, Maxent, and EM
